# Between policy and perception: Stakeholder views on addressing territorial inequality in Europe

**DOI:** 10.12688/openreseurope.19221.2

**Published:** 2025-05-08

**Authors:** Olga Jubany, Zarko Sunderic, Gordana Matkovic, Malin Roiha

**Affiliations:** 1 Departament d'Antropologia Social, Universitat de Barcelona, Barcelona, Spain; 2 Centre for Social Policy, Belgrade, Serbia

**Keywords:** Territorial inequalities; “left-behind places”; governance; policymaking; European regions; qualitative research.

## Abstract

**Background:**

Long-standing territorial disparities have evolved into novel forms of inequality, exacerbated by a decline in social status and the protection afforded to citizens. Territorial inequality extends beyond economic disparities in income and wealth, encompassing unequal access to fundamental rights and opportunities such as essential services, infrastructure, and education. These disparities pose significant challenges to comprehensive socioeconomic development. This paper is part of a broader research project on “left-behindness,” aiming to explore stakeholders' perceptions of the underlying drivers of territorial inequalities, as well as the governance mechanisms and policy tools aimed at mitigating these issues.

**Methods:**

The analysis draws on data from 20 focus groups conducted between November and December 2023, involving 98 national, regional and local stakeholders from seven European countries: Austria, Belgium, Denmark, Greece, Italy, Serbia, and Spain.

**Results:**

The findings reveal a notable disconnect between national-level discourses on territorial inequalities and the priorities of local and regional stakeholders across the seven countries. While territorial disparities are acknowledged within policy frameworks, efforts to address these issues are often impeded by governance challenges, including tensions between centralization and decentralization, fragmented coordination, and insufficient horizontal and vertical cooperation among actors at different levels of government.

**Conclusions:**

The research underscores the necessity of adopting place-sensitive, context-specific approaches to address territorial inequalities. It highlights the need to address demographic challenges, geographic isolation, and inequitable funding mechanisms, particularly in underserved regions. Aligning policy interventions with the diverse and context-dependent challenges faced by “left-behind” areas is essential for the effective mitigation of territorial disparities.

## Plain Language Summary

Long-standing differences between regions have led to new forms of inequality, worsened by declining protections and support for citizens. Territorial inequality isn’t just about differences in income or wealth; it also includes unequal access to basic services, infrastructure, and education, which affect overall development and opportunities.

This paper, part of a larger research project on “left-behind” areas, examines how local and regional stakeholders perceive the causes of territorial inequalities. It also looks at the policies and governance systems designed to reduce these inequalities. The study is based on 20 focus groups with stakeholders from seven European countries: Austria, Belgium, Denmark, Greece, Italy, Serbia, and Spain.

The findings reveal a significant gap between national-level discussions on territorial inequalities and the priorities of local and regional stakeholders. While policy agendas acknowledge these differences, progress is often slowed by governance issues, such as tensions between centralized and decentralized decision-making, poor coordination, and a lack of cooperation between different levels of government.

The research highlights the need for policies that are tailored to local challenges. Solutions must consider demographic changes, geographic isolation, and unfair funding practices, especially in regions that lack essential resources. Overall, the study emphasizes the importance of aligning policies with the specific needs of areas often described as “left-behind.”

## Introduction

The persistence of long-standing disparities between territories – countries, regions and municipalities, has given rise to new expressions of inequalities, exacerbated by a decline in the status and protections afforded to citizens. This territorial inequality is particularly pronounced in regions experiencing socioeconomic stagnation. While national economic growth across European countries may obscure some disparities, these become increasingly evident when examining differences between European regions, where social region-specific decline continues to deepen ( Mehlbye *et al.*, 2019). Since the early 1980s, austerity policies and global economic restructuring significantly contributed to the deepening of these inequalities. This led to a stark polarization, as certain territories experienced economic growth and prosperity, while others suffered persistent losses of income, employment opportunities, and access to essential services ( Alvaredo *et al.*, 2018).

Territorial inequality nowadays is understood as a multifaceted issue that extends beyond economic disparities in income and wealth. It encompasses unequal access to fundamental rights and opportunities, expressed in the lack of essential services, infrastructure, and education, thereby posing broader challenges to all dimensions of socioeconomic development. These inequalities have led to major disparities in wellbeing across critical dimensions such as health, education, and employment, particularly in so called "lagging regions" ( Wuthnow, 2018). Such regions – often rural or post-industrial areas, face compounded challenges intrinsic to economic decline, which are exacerbated by a lack of skilled labour, inadequate infrastructure, and hampered accessibility.

In this context, the concept of "left-behind places", as defined by Isakjee and Lorne (2019), appears particularly relevant to speak about the persistent exclusion of these regions from broader economic growth. With this understanding, we can trace the narrative of contemporary territorial inequalities in Europe back to the financial crisis of 2008, as a turning point of intensifying disparities within countries. As many scholars have shown ( Becker, 2020; Görmar *et al.*, 2019; Kölling, 2021), austerity measures and a shift towards a neoliberal growth agenda in EU cohesion policies following the Lisbon Treaty of 2007, contributed to the solidification of this trend. While discrepancies between EU member states have narrowed, inequality within countries has risen, exacerbating the marginalization of “left-behind places”. These regions' exclusion has fuelled political discontent, reflected in the success of populist, anti-EU parties in areas often referred to as “places that don’t matter” ( Rodríguez-Pose, 2020). Such dynamics heightened awareness of the urgency for more nuanced and place-based policy approaches in EU cohesion policies. However, despite an evident need to develop place-sensitive policy frameworks, there is limited research that addresses how national, regional and local stakeholders perceive governance tools designed to mitigate territorial inequalities.

Drawing on an original qualitative study, this paper addresses this gap by analysing focus group data from seven European countries to explore governance mechanisms and policy drivers influencing territorial inequality. By incorporating perspectives and experiences of stakeholders directly involved in policy implementation, this analysis enhances the alignment of policy interventions with the diverse, context-specific realities of European regions.

## Scholarly perspectives on place-sensitive policy approaches

As exposed by recent debates, development policies aimed at rural, deprived, or "left-behind" areas are often conceived in metropolitan centres, leading to a disconnect between policy design and the actual needs of these areas ( MacKinnon *et al.*, 2022; Pike *et al.*, 2023). As a result, many of these policies are characterised by top-down, growth-oriented strategies that have failed to bring meaningful improvements to the regions they target ( Hadjimichalis & Hudson, 2014; Lang & Görmar, 2019). Despite several EU initiatives aimed at reducing territorial inequalities, many EU-wide policies have been criticized for their broad, one-size-fits-all approach ^1^, which fails to account for the unique needs and opportunities of different regions ( Ezcurra, 2019).

A growing body of literature critiques the neoliberal growth paradigm, which underpins current European regional development policies, as unsuitable for rural or less densely populated areas ( Hadjimichalis & Hudson, 2014; Lang & Görmar, 2019; MacKinnon *et al.*, 2022). These argue that such policies overlook the unique potential of “left-behind” places, favouring competitive export sectors and neglecting key local industries such as the service sector, which plays a crucial role in sustaining household incomes and economic activity in less prosperous regions. Moreover, conventional approaches to addressing territorial inequalities often fail to adopt place-based, endogenous strategies that build on local strengths. Instead, they may inadvertently encourage outmigration from less prosperous areas to economically thriving urban centres, exacerbating feelings of exclusion and disconnection from local communities ( Rodríguez-Pose, 2018).

Such dislocation is particularly harmful for less mobile populations, who may be further marginalized by policies that prioritize metropolitan models of development. As Rodríguez-Pose (2018) emphasizes, the neglect of local identity and the emphasis on mobility contribute to a sense of political disenfranchisement, which in turn fuels dissatisfaction with mainstream political structures. The result is a growing loss of belonging and identity in left-behind regions, alongside a perception of political underrepresentation ( MacKinnon *et al.*, 2022; Rodríguez-Pose, 2018).

EU territorial cohesion policies have also been the subject of criticism for their limited success in addressing territorial inequalities. Although these policies have targeted “left-behind places”, they have largely failed to reduce disparities between regions. Scholars argue that the issue is not necessarily the absence of policies, but the wrong types of policies, which have reinforced the marginality of these areas rather than alleviating it ( Crescenzi *et al.*, 2020; Dijkstra *et al.*, 2020; Tallon, 2021). Research on protest voting in these regions, for instance, suggests that the effectiveness of EU funding is less about the amount of financial support and more about its local impact. Funding that has created long-term employment opportunities has been shown to positively affect communities, reducing the appeal of anti-EU parties. In contrast, the absence of meaningful local impacts has intensified discontent and fuelled populist sentiment ( Dijkstra *et al.*, 2020; Tallon, 2021). The latter affirms that policies operate within a landscape where political and economic dynamics are strongly influenced by place-based effects ( Gordon, 2018). 

However, for policies to effectively address territorial inequalities, it is crucial to gather evidence from local communities that highlights existing gaps and challenges. As Rodríguez-Pose (2018) argues, policy responses must be place-sensitive, rooted in both theoretical frameworks and empirical evidence, while also considering the unique structural opportunities, potential, and constraints of each locality. This paper presents the first step in this process, drawing on focus groups with local and regional stakeholders and analysing the key insights.

## Methods

Studies on territorial inequalities often adopt a quantitative approach, relying on socioeconomic data to analyse patterns, trends, and disparities across regions. While these studies provide valuable insights, such as statistical measurements of income inequality, employment rates, or access to services, they frequently lack the depth needed to capture the lived realities, processes, and complex social dynamics underlying these disparities. This paper adopts a qualitative approach precisely to “uncover the processes and meanings that undergird socio-spatial life” ( Herbert, 2000), offering a deeper understanding of both the subjective and material experiences of inhabiting areas designated as “left behind.” This qualitative lens sheds light on how professionals living in these regions actively work to “re-arrange” conditions to address inequalities. By exploring, through focus group discussions, the perceptions and perspectives of those operating within regions affected by these disparities, the paper exposes how local stakeholders and governance structures navigate challenges, develop strategies, and mobilize resources to mitigate inequalities. This analysis provides a richer and more nuanced understanding of the mechanisms driving territorial inequalities and the potential pathways for alleviating them. Beyond focus groups, the research also incorporates insights from a thorough and critical examination of national and European policy frameworks.

The findings presented in this paper are grounded on focus group discussions conducted in seven European countries—Austria, Belgium, Denmark, Greece, Italy, Serbia, and Spain, selected with an aim for diversity in terms of policy frameworks, historical and socio-economic contexts, as well as geographical distribution. In addition to EU Member States, the sample of countries also include Serbia, which has been a candidate for EU membership since 2012. In each country, two to three specific areas ^1^ were selected at the LAU level (municipalities), based on the following main criteria: First, economic indicators revealing a certain degree of poverty of the local population were considered, mainly related to the EU “ *At risk of poverty or social exclusion*” (AROPE) Indicators. Depending on the availability of data on a local level, these ranged from GDP per capita ^2^ to low levels of disposable income per household, low purchasing power or low levels of taxable incomes. Second, the places were also chosen with respect to the following indicators or life areas, as one main aim of the research was to analyse “left-behindness” beyond an exclusivelyeconomic perspective: 1. Social services and Health: a lack or low quality of basic infrastructure, as well as social and health services is an important indicator of “left behindness”; 2. Education: Lower levels of formal or recognised education was be considered as an indicator of territorial inequality; 3.Employment: the proportion of residents who do not actively participate in the official labour marked was considered a key indicator; 4. Community and social life: looking at indicators such as local population density; access to local activities (sporting and public facilities), youth centres, libraries, and social organizations; crime rates; level of safety, level of trust as well as voting behaviour and/or the right to vote among the local population; 5. Housing: The availability, affordability and accessibility of housing was taken into account, but also the quality of housing space, its location within the chosen locality and in relation to the existing infrastructure, as well as environmental aspects such as contamination; 6. Mobility: The location of a place in relation to public transportation infrastructure, as well as access to public, private and freight transport systems.

### Focus group composition and sample

The focus groups served as a platform to engage with stakeholders with knowledge of the socioeconomic and institutional dynamics that shape territorial inequalities in their respective regions. The groups were designed to explore experiences, perceptions, knowledge, and motivations underlying the selection and implementation of policies and strategies aimed at tackling territorial inequalities. Also, they were aimed at identifying patterns and drivers of these inequalities, particularly from a governance perspective, and examining how policies interact with specific regional contexts. The focus groups were designed to include different levels of policymaking, including local, regional and national levels. Hence, one to two focus groups in each country were conducted in relation to the LAU level areas selected for research. The other focus groups related either to the regional or national level, depending on the relevance of regional frameworks in each country.

A comparative analysis across countries allowed for the identification of variations in policy approaches, highlighting the interplay between national and regional strategies, and uncovering shared challenges and opportunities in addressing territorial disparities. This qualitative and comparative methodology provides critical insights into the complex and multifaceted nature of territorial inequalities and offers a basis for more informed and targeted policy interventions.

Between November and December 2023, 20 focus groups were conducted by research teams in the seven countries, with a total of 98 participants. This was guided by methodological considerations aimed at ensuring both data saturation and validity in the study, aligning with qualitative research best practices ( Guest *et al.*, 2017). Prior studies suggest that code saturation typically occurs within four focus groups, however more groups are needed to fully understand the issues discussed (meaning saturation), with the number depending on the heterogeneity of participants and the complexity of the topic ( Hennink *et al.*, 2019). Each focus group consisted of approximately five participants on average, ensuring both rich, interactive discussions and the inclusion of varied experiences. This balance aligns with recommendations in qualitative research, where smaller, well-structured groups (3–10 participants) foster deeper engagement while avoiding superficial discussions ( Krueger & Casey, 2014), particularly in focus groups with expert participants ( Guest *et al.*, 2017)

In terms of participant profiles, 41.84% of participants were institutional stakeholders with a role in addressing territorial inequalities either at the local, regional or national level, that is, representatives from local, regional, and national institutions, such as municipal authorities, government officials, and policymakers. Another main group of stakeholders involved were representatives from civil society organizations (42.86%), with participants from grassroots movements, non-governmental organizations, and community initiatives. A third group, included in some but not all countries, were academic researchers and experts from related fields (15.31%). In a few groups, where deemed relevant to the local context, participants from the private sector were also included to add perspectives on economic development and local challenges. This diversity of participants facilitated a comprehensive and multi-faceted discussion on the complexities of territorial inequalities in rural, urban and post-industrial contexts. In terms of territorial governance levels, 50% operate at the local level, followed by national (21.43%) and regional (17.35%) levels. A smaller proportion of participants (4.08%) are associated with the EU level in some capacity, in addition to their involvement with institutions at the national or regional level.

The location of the focus group, the areas covered, the number of participants, and the gender distribution within the focus groups are outlined in Table 1.

**Table 1.** Focus group locations and sample.

Focus group code	Country	Location	Number of participants	Women	Men
WP2-AT-FG1	Austria	Jennersdorf	4	0	4
WP2-AT-FG2	Austria	Ternitz	3	1	2
WP2-AT-FG3	Austria	Vienna	4	4	0
WP2-BE-FG1	Belgium	Marchienne-Au-Pont	5	3	2
WP2-BE-FG2	Belgium	Couvin	7	5	2
WP2-DK-FG1	Denmark	Frederikshavn	4	2	2
WP2-DK-FG2	Denmark	Morsø	6	3	3
WP2-DK-FG3	Denmark	Online, with participants from Copenhagen, Lemvig, Silkeborg, Aalborg	4	1	3
WP2-EL-FG1	Greece	Pyrgos	7	2	5
WP2-EL-FG2	Greece	Athens	6	4	2
WP2-IT-FG1	Italy	Veneto	4	3	1
WP2-IT-FG2	Italy	Veneto	5	1	4
WP2-IT-FG3	Italy	Venezia	7	4	3
WP2-IT-FG4	Italy	Sardinia	6	5	1
WP2-RS-FG2	Serbia	Golubac	5	3	2
WP2-RS-FG3	Serbia	Belgrade	3	1	2
WP2-RS-FG4	Serbia	Belgrade	5	3	2
WP2-RS-FG1	Serbia	Surdulica	5	1	4
WP2-ES-FG1	Spain	Barcelona	5	2	3
WP2-ES-FG2	Spain	Catalan Pyrenees	3	2	1
**TOTAL**			**98**	**50**	**48**

The focus group discussions were based on a set of common themes ^3^ across the seven countries ( Universitat de Barcelona & Centre for Social Policy, 2025), with discussions led by one or two experienced facilitators per group..

### Data management and analysis

All the focus groups were recorded with a voice recorder and were subsequently transcribed. These transcripts were then imported into a software tool for qualitative analysis. Based on a grounded analysis approach, the exploration and data analysis followed an open coding process and a line-by-line reading of the data, assigning initial codes. The comparative analysis of the data followed the same pattern, creating more nuanced distinctions where necessary. Codes were then grouped into broader categories based on their conceptual similarities, and themes were identified through an axial coding process ( Universitat de Barcelona *et al.*, 2024).

### Ethics statement

Ethical approval for this research was sought and received by the Comisión de Bioética de la Universidad de Barcelona (CBUB - Institutional Review Board IRB00003099), on July 20, 2023.

Common ethical guidelines and standard processes were established for all focus groups, including standardized informed consent sheets. All participants provided their written informed consent, including permission to record data.

To protect the anonymity and confidentiality of the focus group participants, and to facilitate data management, each participant was assigned a code, following the pattern <Task number-Country-Focus group number-Participant number>. As it is necessary to be able to re-trace a participant's identity to fully respect the right to withdraw from the study, in this context it is more relevant to talk about “pseudonymisation” than of full anonymisation. Additionally, any information that might identify a participant was strictly excluded from the analysis process.

## Results and analysis: from concepts to experiences and impacts

In the following sections, the findings from the empirical research are presented and analysed, with a particular focus on the perceived drivers of territorial inequalities identified through the qualitative research process. These drivers primarily concern governance mechanisms, such as fragmented policy frameworks, insufficient collaboration and inter-agency cooperation, inadequate funding schemes for local levels, and the balance between policy centralization and decentralization. Additional factors include limited institutional capacities, depopulation, and geographical challenges. Before delving into these findings, however, the concept of “left-behindness” is examined in relation to other concepts used in national, regional, and local policy-making, drawing on insights from the empirical research.

### “Left-behindness” in territorial inequality discussions

One of the central insights of this research is the significance of the term “left-behindness” in framing debates on territorial inequalities. These terms go beyond describing regional disparities, drawing attention to broader issues of conceptualisation of development, exclusion, and spatial injustice. By exploring their use, the research reveals how such language influences the understanding and discussion of territorial inequities. However, research revealed that, regardless of its prominence in policy debate, this term is scarcely used across all the countries both in political and academic discourse. Instead, participants tend to prefer expressions such as “marginality”, “remoteness”, “disadvantage”, “underdeveloped areas”, and “rural areas”. Similarly, the term “left-behind places” is not widely utilized in country-specific discussions and its translations vary. Across the different countries, alternatives include concepts such as “inner areas”, “depressed areas”, “marginal areas”, “mountain areas”, “fragile areas”, “peripheral areas”, “deindustrialized areas”, “coastal areas”, or “sacrifice zones”.

The results also underscored that the level of connectedness of an area to other locations plays a pivotal role in defining the area with the concept of “left-behindness”. To illustrate, there is a significant contrast between experiencing a post-industrial scenario in a rural, remote, and declining context, as observed in Morsøe (Denmark), and encountering a post-industrial situation near a well-connected city, exemplified by Murano's proximity to Venice (Italy).

When examining the use of "left-behindness" in policy and public discourse as a concept that relates to territorial inequality, the research revealed that, although desk research on policy documents and grey literature from the seven countries suggests territorial disparities are a prominent concern in national political discourse, there is a notable disconnect between this acknowledgment and the actual prioritization of territorial inequality within policy and public discussions. To nuance this generalized perception, in Italy, participants recognized that territorial inequalities are on the policy agenda but noted that the discussions tend to be ideologically driven and lack a comprehensive perspective. Similarly, in Belgium, while territorial inequality is acknowledged, respondents highlighted the absence of concrete proposals, difficulties in implementation, and challenges in conducting reliable policy evaluations. The conversation is often framed as a regional confrontation rather than a collective reflection on the inequalities between sub-regions. In contrast, participants in Austria perceived that territorial inequality in general is not a major issue in their country. Nevertheless, when discussing differences between regions, the federalist orientation of regional policy was perceived as a driving force behind regional disparities. Different regional policy strategies between federal states, along with regulatory differences—such as in tourism or youth protection—result in a large number of internal borders. These are perceived very strongly in everyday life and are experienced as restrictive. It should be noted here that Austria, a country with around nine million inhabitants, is divided into nine different states. In addition, the country borders eight neighbouring states. This results in a multiplicity of regulatory borders, which gives places that are located between several of these internal and external borders an entangled peripheral location.

### Absence of a policy framework and fragmented competencies

A key finding across all the countries was the lack of a structured and cohesive policy framework to effectively tackle territorial inequality in a systematic way. This absence points to a critical gap in addressing the underlying causes and persistent disparities between regions. Such gap, coupled with a short-term perspective in policy implementation, emerged as one of the main drivers of territorial inequalities in some of the countries, a concern notably observed in Italy, Serbia and Spain: “Allocating resources is not enough. It’s crucial to provide all the necessary conditions to overcome marginalization” (WP2-IT-FG1-P2). In this regard, the absence of a well-organized framework in Italy to streamline all actions, including those related to Cohesion Policy, poses a significant challenge. This has led to a deficiency in comprehensive strategies to effectively address territorial inequalities and bridge existing divides. Furthermore, there is a notable lack of national-level policy evaluations, specifically in terms of their economic implications and outcomes.

Similar to this, findings reveal that Serbia lacks a dedicated institution to coordinate local self-governments, align policies, and support implementation of policies. The country lacks a regionalization policy and corresponding structures. Linked to this, the individual municipal plans, developed in isolation, are perceived as inadequate as a foundation for comprehensive regional development.

Fragmented competencies and a lack of vertical policy coordination between different levels of government also contribute to inter-territorial competition, exacerbating challenges such as mobility: "There are examples of inter-municipal cooperation in our country, but collaboration is not promoted as a development tool from the national level, and there are no incentives provided to encourage the implementation of such cooperation" (WP2-RS-FG3-P3).

The divided powers across different government levels, as strongly observed by participants in Belgium, obstruct the development of a comprehensive vision. A common theme is the dysfunctional link between all government levels, which reinforces coordination challenges between federal, regional, and local levels. In this regard, there is an undisputed assessment in Belgium among participants regarding the need for a comprehensive review of the management of territorial inequalities. They emphasize the importance of a collaborative approach involving local actors, enhanced coordination, citizen participation, and the strengthening of local initiatives for sustainable and socially just economic development.

Overall, the study revealed an inadequate coordination between national, regional, and local levels, highlighting this as the greatest challenge in implementing necessary measures to reduce these inequalities, particularly shown in the case of Spain. The lack of cooperation between urban municipalities and between different levels of governance in Spain exacerbates the challenges faced by urban areas, particularly in terms of extreme vulnerability such as areas of shantytowns (“barraquismo”). In this regard, participants highlight the need for both inter-municipal coordination and supra-municipal coordination as well as a need to establish connections across different governance levels. To achieve this, inclusive dialogue and the active involvement of local-level stakeholders in the policy-making process are perceived as essential.

### Weak collaboration and inter-agency cooperation

Adding to the absence of a policy framework and fragmented competencies, the study also found that territorial inequalities in many countries are significantly driven by weak collaboration among public institutions, limited engagement with civil society, and a lack of horizontal cooperation. This issue is evident across various countries, where fragmented efforts and poor coordination exacerbate disparities. In Greece participants emphasized the need for multi-agency approaches that actively engage citizens and civil society to address territorial inequalities. Further, respondents in both Greece and Denmark highlighted a common problem: national policies tend to prioritize economic growth, often overlooking crucial issues such as access to social services, educational inequalities, employment opportunities, housing challenges, inadequate infrastructure, and digital exclusion. In Denmark, efforts to address these inequalities are particularly hampered by insufficient coordination among institutions.

A similar pattern is illustrated in the case of Spain, where increased collaboration across municipal borders is viewed as essential for tackling inequalities in both rural and urban areas. However, mistrust and conflicting political interests often lead city councils and smaller regions to pursue individual strategies instead of horizontal cooperation. This lack of cooperation fragments initiatives and undermines collective efforts. The presence of institutions and civil society in local communities, on the other hand, is deemed to play a vital role in raising awareness of inhabitants' needs.

In Belgium, civil society organizations face their own struggles with collaboration. Competitive project calls, instead of fostering cooperation, create rivalry among organizations, limiting their ability to pool resources and work together. This competitive environment reduces their collective impact and further impedes efforts to address territorial inequalities.

### Inadequate funding systems and schemes targeting the local level

Inadequate funding is revealed as critical in all cases across Europe. In most of the countries, small municipalities are required to fulfil the same obligations as larger administrative units but with significantly fewer resources available for certain municipal functions. This is particularly the case in Denmark, Italy, Austria, Serbia. However, it is not merely an issue of the absolute difference in budget size between small and large municipalities. The primary challenge lies in the fact that administrative costs and fixed expenses in the budget structure are substantially higher in small municipalities due to significant fixed costs. As pointed out by a participant in Austria: “This distribution of funds to the municipalities according to this scheme is no longer right and it has to be thought differently” (WP2-AT-FG2-P2). Several participants called for a different funding system for municipalities tailored to their local needs and the challenges they face. Respondents emphasize that, for certain functions in small municipalities, more funds should be allocated from the national budget.

Additionally, some national policies and interventions in most observed countries rely on per capita funding. Governments use per-capita funding schemes for certain services in their countries for several reasons. First, it gives the impression of equity and fairness since everyone receives a relatively equal share of the allocated resources, regardless of the size or characteristics of the region or community. It also promotes predictability, efficiency and transparency in funding and helps automatically adjust to changes in communities. Per-capita funding often simplifies administrative processes and reduces the need for complex formulas or assessments to determine funding allocations. However, while per-capita funding has its advantages, it may not be suitable for all services or circumstances. Some services may require more nuanced funding approaches based on specific needs, geographic factors, or socioeconomic considerations. In practice, per-capita funding prioritizes efficiency and thus may lead to actions such as school mergers and closures. The lack of these services, in turn, contributes to population decline in certain areas, resulting in a self-reinforcing cycle of depopulation, aging, and diminished services and social life.

Participants in Belgium also note that public investment heavily favours large cities, leaving peripheral and rural areas lacking resources and infrastructure, contributing to a feeling of “left-behindness”. Even initiatives like the Walloon Economic Recovery Plan, including the Catch Turbo 2.0 plan for the Charleroi Métropole, and other policies of major cities fail to consider local disparities. Consequently, there is a strong perception that areas outside of large cities are neglected, leading them to compete for attention and resources. The feeling of being neglected by public investment and the prioritization of large cities over peripheral areas, as mentioned by Belgian participants further supports the idea that a lack of attention to the specific needs of these places can erode place attachment and contribute to feelings of being left behind. Similarly, the Regulation on the Underdevelopment of Municipalities in Serbia, governing the distribution of funds from the national budget, has remained unchanged since 2014. Consequently, certain impoverished municipalities receive insufficient funding, while wealthier ones continue to benefit, disrupting the intended balance of development funds. The problem of unfair fund distribution goes beyond these transfers, affecting financing and other services that use the rules outlined in the existing Government's Regulation on the Underdevelopment of Municipalities. As one Serbian participant puts it: "May the national authorities grant us the freedom to utilize our resources and enjoy the revenues that legally belong to us. We require nothing more" (WP2-RS-FG1-P3). That is, the Regulation, initially designed for equalization and fair fund distribution, is perceived as intensifying existing disparities, thus undermining trust.

Participants operating at the national level in Austria, on the other hand, emphasize the availability of numerous funding programs designed to address issues of territorial inequality. However, as per the guidelines for applying to these funds, access is limited to municipalities with adequate staff, knowledge, and skills to pursue funding opportunities or projects. Experience often shows that while municipalities with sufficient capacities and competences benefit from these programs, the absence of such capabilities in certain municipalities worsens territorial inequalities. Like this example, bureaucratic difficulties, including extensive paperwork and lengthy processes for funding requests from the regional or provincial government are highlighted as significant governance challenges within rural areas of Catalonia (Spain). Participants perceive a pressing need to modify the rules for distributing existing funds allocated to small and underdeveloped municipalities and to establish additional intermediary mechanisms to support the application and implementation of such programs.

Another challenge revealed is the lack of enforcement of regulations in Serbia, which creates uncertainty in accessing the resources guaranteed by existing legislation. This includes financial, natural, and human capital resources in certain municipalities. To address financial uncertainty and to secure resources necessary for their regular functioning and development, some municipalities rely on establishing strong political connections between their local leadership and decision-makers at the national government level. However, this practice undermines trust in institutional effectiveness and reinforces perceptions of favouritism and inequity. Summarizing this sentiment, a participant in Serbia stated that "The application of laws and their interpretation is weak and selective. A law applies to some but not to others. That's the problem" (WP2-RS-FG1-P3).

### Between policy centralization and decentralization

Left-behind places emerge in the tension between processes of (de)centralization within countries. Both centralization and decentralization have the potential to foster territorial inequalities, and both approaches may prove ineffective and inefficient in addressing and combating these disparities. It is thus crucial for countries to strike the right balance between policy centralization and decentralization, considering evolving needs and historical circumstances.

Centralization can result in uneven distribution of resources, economic imbalances, and inadequate attention to the specific needs of various territories, thus leading to increased inequalities. When decision-making and resource allocation are concentrated in a central authority, regions or areas that are not prioritized may experience neglect and disparities in development opportunities, as observed in several countries. Findings in Denmark highlight a socio-geographical imbalance resulting from the significant centralization of the welfare state, triggered by the municipal reform in 2007, and a growing concentration of employment, economic activity, and growth around major cities. After Denmark's municipal reform and the elimination of counties at an administrative level, regional development priorities shifted from territorial equity to a stronger focus on business growth. Findings reveal that the main challenge nowadays is the clustering of private businesses in the country’s two largest cities, Copenhagen and Aarhus. This concentration poses a major obstacle to resource distribution across the country, with only 10% of job growth occurring outside these urban areas. The municipal reform further led to a reduction in jobs suitable for academics and individuals with a high educational level, coupled with more young people moving to major cities for education and work. The challenge lies in encouraging young graduates to relocate to peripheral areas with limited commuting options, as job growth, innovation, and economic opportunities are predominantly concentrated in urban centres. In Serbia, the reluctance to establish some level of regionalization in the country is mainly driven by political considerations. As a result of such circumstances there has been a visible trend toward centralizing resources and decision-making over the last years. Participants from Serbian municipalities underscore that this shift has translated into a loss of local control over substantial resources, including financial, administrative, and human capital. The ongoing centralization presents challenges for municipalities, diminishing their influence at a local level.

Nevertheless, decentralization can also contribute to territorial inequalities, as proved in other countries. Contrary to the previously exposed challenge of dealing with centralization and its effects on the increased territorial inequality, the example from Austria shows how a strong federalist orientation might lead to similar challenges. When decision-making authority and resources are devolved to lower levels of government or local authorities, regions with weaker administrative capacity or insufficient resources may struggle to address their development needs effectively. In some cases, decentralization can lead to disparities in service delivery, economic development, and infrastructure investment, reinforcing territorial inequalities. The effectiveness of decentralization in reducing or exacerbating territorial inequalities depends on the implementation, capacity, and local governance structures.

Results from the Austrian case stress the issue of different regional policy strategies between federal states, as well as regulatory differences in those states, resulting in many diverse rules between neighbouring places and municipalities, creating a sense of internal borders. This leads to a fragmented territoriality, as shown in the case of the municipality of Jennersdorf (Austria), illustrating that regions interconnected in everyday life are not considered as such in terms of regional policy. Thus, the current administrative borders of the region do not align with the "lived and daily experienced region", as "[...] it's almost easier to do proper projects with Slovenia, Hungary, than with Styria, Lower Austria [two federal states]" (WP2-AT-FG1-P1).

The same is revealed in the Belgian case, where there is a lack of coordination between various government levels in an increasingly federalized country. In contrast to the existing trend of federalization, a very small minority calls for a return to stronger federal powers to reduce economic disparities between regions. They question the principle of financial autonomy granted to the regions, arguing that it exacerbates territorial inequalities rather than achieving a fairer distribution of wealth. However, most respondents are still supporting federalisation. To tackle territorial disparities, they call for better coordination between the different levels of government and the measures they adopt. There is also a call for better coordination between regional and local actors, whether by public authorities or civil society organizations. In addition, participants in Belgium state that the principles and sensitivities of political representatives and their parties have a significant influence on the choice of policy strategies for the development of “left behind” areas.

### Lack of institutional capacities

Another critical finding highlights the challenges posed by the limited institutional capacities of smaller municipalities across all countries studied. In both Italy and Serbia, the minimal number of employees in small and underdeveloped municipalities results in significantly heavier workloads for staff compared to their counterparts in larger, better-resourced municipalities. This disparity underscores the strain on local governance and the broader implications for service delivery and regional development. Employees in small municipalities are burdened with diverse professional and administrative duties mandated by regulations from various sectors. As emphasized in the findings from Italy, "the rationale and bureaucracy of the local government budget are the same regardless of the size of the municipality. However, in small municipalities, the personnel may not have all the competencies for fulfilling all necessary requirements” (WP2-IT-FG4-P6).

Another phenomenon revealed as determining in impact is a continuous outflow of skilled professionals in small and underdeveloped municipalities, as particularly observed in Serbia. This diminishes the potential for optimizing available opportunities. Additionally, the ban on hiring in public administration and poor personnel policies further negatively impact the position of underdeveloped areas in Serbia. Weak human resources have a detrimental impact on the delivery of quality services, and the untapped potential for development remains underutilized. Even applying for much needed funding, whether regional, national or European, becomes problematic in this setting, as both know-how and human resources may be lacking.

Municipalities highlight their insufficient resources to hire skilled professionals such as IT specialists, civil engineers, experienced lawyers, and public procurement experts. A Danish participant underscored that “We also require lawyers in our municipality, just as larger municipalities do” (WP2-DK-FG2). The challenge arises from the inability to attract these professionals, often due to the comparatively low salaries offered by municipalities. Additionally, the overall quality of life and limited opportunities prompt skilled professionals to migrate to larger cities. These observations are consistently reported across all seven countries.

### Population decline and shifting demographics

Demographic decline and changes in population structure are perceived as additional challenges and thus as calling for new roles for the state. The increasing number of elderly individuals, coupled with declining birth rates, and the emigration of the working-age population to larger, more developed areas, threatens the uniform provision of welfare services. Unfavourable demographic trends not only reduce the municipality's economic capacity to meet the growing needs for dependent services sustainably but also diminish the demand for certain services due to a declining younger population. This, in turn, renders the provision of essential services too costly. Locally based services such as schools, daycare centres, doctors, dentists, etc., are under pressure. These phenomena are evident in all seven countries. The population size decrease threatens the vitality of municipalities, hampering efforts to drive local development.

In this regard, participants in Austria highlighted the absence of an overarching strategy to address demographic decline. There is a recognized need for a comprehensive plan at the national and federal state levels to attract young people, labour force, and immigrants to small communities and cities. Some participants believe that the absence of such a plan penalizes smaller and more peripheral municipalities.

In Greece there is a significant concern over the phenomenon of brain drain, wherein young people migrate to major cities, causing the closure of factories, industries, and local crafts. This migration significantly impacts the productivity at the local level. A specific case highlighting this issue is the Municipality of Acharnes, which is portrayed as lacking adequate support from the central government for its development programs. This lack of support contributes to a sense of unfair downgrading within the municipality. The consequences of this brain drain are far-reaching, affecting not only the local economy but also the overall development prospects of the region.

In depopulated areas, the overall population is decreasing, leading to a reduced number of students in schools. This decline in student enrolment makes it financially challenging to sustain and operate educational facilities. As observed in the municipality in Serbia (Golubac), a high school was established a few years ago with funding support from the central Government, with the aim of retaining young residents by providing quality education. However, after seven years, only one class in the 4th grade of high school remains, despite the municipality's efforts to alleviate education costs for families, including covering transportation and school textbooks.

In response to this phenomenon, Denmark has implemented policies focused on both educational institutions and the localization of public administration, placing them at the centre of efforts to alleviate the consequences of territorial inequality. The rationale behind this approach is twofold. Firstly, the relocation of parts of the central public administration to less developed areas aims to boost demand and create more white-collar jobs in the peripheral regions of the country. Secondly, this initiative seeks to retain the younger population by providing opportunities for higher education in these areas.

### Geographical disparities in quality of life

Topography and geographical positioning are also factors that significantly shape the quality of life and may create inequalities if not addressed sufficiently. This relates particularly to remote mountain regions (Italy, Austria and Spain), islands (Greece) or some border areas (Serbia, Austria). Difficult terrains, such as remote mountainous areas or islands, frequently face challenges related to limited accessibility caused by inadequate transportation infrastructure. In this regard, geographical positioning can restrict residents' ability to reach essential services, employment opportunities, and educational facilities. The latter also affects infrastructure development contributing to disparities between regions. Similarly, economic opportunities, housing availability, and property values are influenced by topography, impacting arable land, tourist areas, and housing space.

In Italy, territorial inequalities are historically influenced by the north-south divide and the so-called "southern question," reflecting lower economic development in the south compared to the more industrialized north. In the last two decades, academic and policy debates foregrounded a more complex picture of territorial inequalities in Italy, displaying an archipelagic distribution of territorial inequalities, with marginalised conditions persisting even in the metropolitan poles or wealthy areas and in the so-called inner areas. Further, some Italian inner areas suffer from depopulation and ageing despite being economically wealthy. This is due to the “peripherality from the essential services, including healthcare, school and education, transportation services” (WP2-IT-FG4-P5). The impact of remoteness extends beyond economic considerations, influencing individual and household choices even in economically prosperous areas. Participants in Italy emphasized the importance of access to services in determining the attractiveness of a territory for employment, highlighting a clear correlation (WP2-IT-FG2-P3).

Likewise, territorial inequality in Serbia also has historical roots, prominently seen in the disparities between the developed north and impoverished south (WP2-RS-FG3-P3). The impact of the former socialist/communist regime further complicated the issue with the establishment of industrial complexes under a "planned economy". The dissolution of these structures in the '90s, coupled with challenges during the privatization process, particularly affected the southern regions, leaving them without a solid foundation.

Nevertheless, establishing a clear relationship between a community’s administrative ties to an urban centre and ability to benefit from the development of that urban area is often challenging. For instance, in places like Murano, "local communities lag behind as the municipal government prioritizes the interests of specific groups and economic entities rather than addressing the unique needs of the territory" (WP2-IT-FG3-P1). The presence of industrial estates in peripheral areas of Spain also poses isolating factors. Even the demographic composition of an area, particularly in terms of ethnicity, can lead decision-makers to overlook a specific place and neglect public investments or service provision. Neighbourhoods such as La Mina (Barcelona, Spain), characterized by a significant Roma population, are often deemed isolated enclaves, mainly due to a perceived lack of political interest in improvement.

### EU funding disparities as driver of territorial inequality

An important final insight from the research is the recognition that disparities in EU funding are widely perceived as a key factor driving territorial inequalities in the studied countries. Empirical findings point towards a disadvantage of places in Austria compared to Hungary due to higher EU funding for Hungarian businesses close to the border. Businesses tend to select their locations based on areas with higher EU funding, resulting in perceived competitive imbalances on the Austrian side of the border. Austrian participants also express dissatisfaction with the LEADER program, particularly in terms of the federal state's strategy and EU bureaucratic requirements.

In Denmark, there are also concerns about how EU funds are distributed, specifically due to the reliance on regional GDP assessments. Experts on territorial inequalities in Denmark, for instance, show significant concern around the distribution of EU funds to different regions in Denmark. The current allocation is based on regional GDP assessments, where Region Zealand has the lowest GDP. However, this indicator masks the reality that many residents of Region Zealand work in Copenhagen, contributing significantly to Copenhagen's GDP and only marginally to Region Zealand's GDP. This results in substantial funds being allocated to a region with less actual need, compared to areas that are further away from metropolitan areas. Despite comparable GDP levels, these remote areas experience a lower standard of living than Region Zealand, which benefits from its proximity to Copenhagen and EU regional support programs. Further, Danish participants mentioned that Denmark may not be fully capitalizing on EU opportunities, particularly in relation to the EU's innovation support for large companies. Economic resources, knowledge, and capacities need to reach rural areas for development beyond relying solely on blue-collar workers and tourism.

Also in Italy EU policies and funds are recognized as critical for addressing territorial inequalities. Italian participants emphasized the unique characteristics of EU policies compared to domestic Italian policies, emphasizing their structured and continuous nature. A key strength of EU policies is their continuity, extending not only throughout the seven-year programming cycle but also across successive cycles. This sustained approach highlights the necessity of long-term interventions to effectively address critical challenges. Securing funding for each seven-year European programming cycle is essential to preserve consistency and maximize the impact of these initiatives over time.

## Conclusions

The data discussed throughout this paper underscore a critical disconnect between national-level discourse on territorial inequalities and priorities identified by local and regional stakeholders across the seven countries studied. While territorial inequalities are increasingly acknowledged in policy agendas, progress is constrained by ideological framing, the absence of actionable proposals, and significant implementation challenges. This highlights a persistent gap between the design of policies and the structural realities faced by underserved regions, suggesting the need for more pragmatic and context-sensitive approaches to address these disparities effectively. Territorial inequalities represent a multidimensional challenge that requires a comprehensive policy framework incorporating both short-term and long-term perspectives. National policy frameworks should not solely prioritize economic growth but also address critical issues such as access to social services, educational inequalities, employment opportunities, housing challenges, inadequate infrastructure, and digital exclusion. Ensuring well-being and access to essential services, regardless of the level of development, suggests incorporating policies such as fiscal equalization into these strategic documents. Considering the tendency of governments at all levels to operate within short-term policy horizons, it is advised that any major policy deviations—particularly those misaligned with established long-term strategic frameworks—be substantiated through rigorous justification and inclusive stakeholder consultations. This entails concerted efforts to include the perspectives of residents from “left-behind areas” in consultation processes during the development of policy and strategic documents.

The findings also show the relevance of governance structure as a primary driver of territorial disparities, with tensions between centralization and decentralization significantly shaping unequal resource distribution. Centralized systems often marginalize peripheral regions, while decentralized frameworks may exacerbate inequalities when local authorities lack the administrative capacity or resources to effectively address their development needs. Fragmented governance, inadequate intergovernmental coordination, and insufficient involvement of local stakeholders may hinder cohesive strategies to reduce territorial inequalities. As highlighted by Rodriguez-Pose (2018), “one of the main challenges for the implementation of this type of place-sensitive policies in lagging-behind and/or declining areas is institutional”. Striking the right balance between centralization and decentralization is one of the key prerequisites for reducing territorial inequalities. In addition to mechanisms for coordination and cooperation, as well as enhancing capacity building and financial support for territories “left behind”, measures may also involve harmonizing regulatory frameworks where appropriate, reforming national-level monitoring and equalization systems, and defining shared governance models—such as co-decision or co-financing—for major projects. As supported by the empirical data presented, to address these challenges, a place-sensitive approach that fosters cooperation between local and supra-municipal actors is essential.. Addressing the lack of coordination between national, regional, and local levels, along with insufficient intersectoral and inter-agency cooperation, is one of the most challenging tasks. Recommendations in this area need to be country-specific as they necessarily depend on the structure and organization of the system, particularly regarding the degree of decentralization and the division of competencies in specific policy areas. In highly decentralized countries and in cases of fragmented competencies, it is essential to strengthen a comprehensive vision, through cooperation and coordination. In centralized countries, institutions and mechanisms should be established to coordinate local self-governments at the regional level, align policies, and ensure the effective policy implementation. These findings echo the development approach suggested by MacKinnon *et al.* (2022), which builds on local assets, resources, and social practices. This necessitates bottom-up approaches and the involvement of local actors in shaping their development. Integrating local voices into policy-making and promoting multi-agency collaborations, particularly with civil society, can aid in developing more responsive, context-specific solutions.

Strengthening local capacities through continuous training, targeted support mechanisms, and inter-municipal knowledge exchange is crucial for building more resilient governance structures in most countries. Empirical evidence presented in the article underscores the importance of targeted capacity-building interventions, particularly those that support participatory planning and enable local actors to navigate complex policy frameworks. It is important to design innovative mechanisms for including the perspectives and concerns of marginalized groups, and to train local governments and communities to effectively implement these mechanisms. In some countries, the establishment of inter-municipal platforms for knowledge and experience exchange is especially vital, not only to share best practices but also to discuss unsuccessful (failed) practices, with mandatory analytical summaries of the factors contributing to the outcomes. A combination of policies focusing on capacity building, workforce incentives, inter-municipal cooperation, and improved access to funding can help address the institutional capacity gap in smaller municipalities.

Moreover, as has been exposed, systemic demographic challenges faced by smaller municipalities are critical in generating territorial inequalities. These areas, with limited resources and substantial service obligations, are caught in cycles of depopulation, aging populations, and deteriorating infrastructure. Per capita funding models often fail to account for the unique needs of these municipalities, leaving them unable to compete for skilled professionals or provide essential services. Broad, efficiency-driven approaches may not address the specific challenges of “left behind”' places with declining populations and service needs, echoing the criticism by MacKinnon *et al.* (2022) of policies that fail to cultivate the neglected human potential of these areas. Addressing these challenges requires a comprehensive strategy to address population decline and shifting demographics, highlighting the importance of an overarching plan at regional, federal, and state levels combining strategies to attract and retain residents, strengthen local economies, improve infrastructure, prevent brain drain and ensure access to essential welfare services. A similar approach is also necessary to respond to the challenges faced by residents of remote or challenging terrains. Geographical factors, such as isolation in remote mountain areas or proximity to border regions, further exacerbate disparities between territories. Policies must be more attuned to these unique geographic challenges to improve residents' quality of life and foster economic development. Moreover, the allocation of EU funds has the potential to exacerbate territorial inequalities, particularly in border regions, if not carefully managed. Addressing EU funding imbalances should ensure fair competition and avoid distortions through targeted interventions and policy adjustments. Based on the research conducted, two specific recommendations stand out. First, in border regions experiencing funding disparities, policies should promote cross-border cooperation between neighbouring countries, facilitating joint investments and shared projects that tackle common challenges. This would reduce competitive imbalances and foster mutual growth. Second, the reliance on regional GDP assessments for EU funding distribution should be reassessed. A more comprehensive approach could ensure that resources are directed toward regions with genuine need, recognizing that GDP alone may not fully capture the economic and social conditions on the ground.

Finally, there is an urgent need for systematic investment in research and policy evaluation specifically targeting territorial inequality. At present, monitoring and evaluation mechanisms for strategies addressing left-behind areas remain limited, with many policies never formally assessed. Although strategy templates often include defined indicators for monitoring, these are either not consistently applied or the results are not made publicly available in some countries. Before developing new strategic documents, it is essential to conduct transparent reviews of the implementation and outcomes of previous ones. Above all, governments must prioritize sustained and structured evaluation efforts to better understand and address territorial disparities. It is also crucial to assess the impact of national space-blind policies on left-behind areas as they may be less effective due to design, implementation, or non-take-up issues.

Grounded on contemporary empirical evidence, this paper has emphasized the urgent need for comprehensive, place-based policies that prioritize local engagement, cross-sector collaboration, and multi-level governance. To conclude, it is important to acknowledge several limitations of this study. First, while the focus group participants were carefully and strategically selected to ensure a degree of diversity, the sample is not representative of the broader population or of all relevant stakeholders involved in territorial governance. Moreover, the recruitment of participants primarily from institutional and civil society sectors may have introduced a degree of selection bias, potentially excluding more marginalized voices or those less directly engaged in policy-related processes. Nevertheless, these perspectives have been explored through ethnographic fieldwork and analysed in forthcoming publications. Second, although the selection of seven countries aimed to capture a range of geographical and political contexts across Europe, the number of LAU-level areas studied within each country was necessarily limited. Certain regional dynamics or national policy approaches may therefore not be fully captured. Finally, the uneven relevance and role of regional governance frameworks across the participating countries influenced both the composition of the focus groups and the types of insights generated. This variability may affect the comparability of findings related to regional policy coordination and implementation, especially where regional structures are weaker or less institutionally embedded.

## Ethics and consent: 19221

Ethical approval for this research was sought and received by the Comisión de Bioética de la Universidad de Barcelona (CBUB - Institutional Review Board IRB00003099), on July 20, 2023. All participants provided their written informed consent, including permission to record data.

## Data Availability

Zenodo repository_- Focus groups with local stakeholders and policymakers on drivers and policies of territorial inequalities https://doi.org/10.5281/zenodo.14094939 ( Universitat de Barcelona *et al.*, 2024). This dataset consists of focus group data generated in WP2 of the EXIT project in seven countries: Austria, Belgium, Denmark, Greece, Italy, Serbia, Spain. The aim was to analyse local policymakers' and other relevant stakeholders' perceptions of policy drivers and approaches to territorial inequalities. The dataset contains factsheets from 20 focus groups. To protect personal data and pseudonymised identities, access to this data set is restricted. Data access may be obtained by submitting a request to the research coordinator Olga Jubany ( olga.jubany@ub.edu) at University of Barcelona, providing written plans and justification for what is proposed with the data. Requests will be reviewed by the research coordinator University of Barcelona together with the task-leader Centre for Social Policy, in consultation with the project’s Ethical Standards Committee. Data are available under the terms of Creative Commons Attribution 4.0 International. Zenodo Repository- EXIT_WP2_Focus groups_Guiding questions https://doi.org/10.5281/zenodo.14726432 ( Universitat de Barcelona & Centre for Social Policy, 2025). This dataset contains following extended data: - EXIT_WP2_Focus groups_Extended data_Guiding questions.txt Data are available under the terms of Creative Commons Attribution 4.0 International

## Footnotes

^1^Such as the Cohesion Fund, see https://ec.europa.eu/regional_policy/funding/cohesion-fund_en.
