# Supplementary material for: Between policy and perception: Stakeholder views on addressing territorial inequality in Europe
Source: Open Res Eur. 2025 May 8;5:40. Originally published 2025 Feb 4. [Version 2] doi: 10.12688/openreseurope.19221.2 (PMC12120432; doi:10.12688/openreseurope.19221.2)
Supplement: Peer review history

# Reviewer report: Reviewer response for version 2

**DOI:** 10.21956/openreseurope.21853.r54002

**Published:** 2025-06-09

**Authors:** Ray Hudson

**Affiliations:** 1 University of Durham, England, UK

I recommend the acceptance of this manuscript.

If applicable, is the statistical analysis and its interpretation appropriate?

Not applicable

Is the study design appropriate and is the work technically sound?

Partly

Is the work clearly and accurately presented and does it engage with the current literature?

Partly

Are the conclusions drawn adequately supported by the results?

Partly

Are sufficient details of methods and analysis provided to allow replication by others?

Partly

Are all the source data and materials underlying the results available?

Partly

Reviewer Expertise:

NA

I confirm that I have read this submission and believe that I have an appropriate level of expertise to confirm that it is of an acceptable scientific standard.

# Reviewer report: Reviewer response for version 2

**DOI:** 10.21956/openreseurope.21853.r54003

**Published:** 2025-06-02

**Authors:** María Mercedes Di Virgilio

**Affiliations:** 1 Instituto de Investigaciones Gino Germani, University of Buenos Aires | CONICET, Ciudad Autonoma de Buenos Aires, Ciudad Autónoma de Buenos Aires, Argentina

I have no further comments to make. The revised article sets the comments.

If applicable, is the statistical analysis and its interpretation appropriate?

Not applicable

Is the study design appropriate and is the work technically sound?

Partly

Is the work clearly and accurately presented and does it engage with the current literature?

Partly

Are the conclusions drawn adequately supported by the results?

Yes

Are sufficient details of methods and analysis provided to allow replication by others?

No

Are all the source data and materials underlying the results available?

Yes

Reviewer Expertise:

Public policies, urban inequalities.

I confirm that I have read this submission and believe that I have an appropriate level of expertise to confirm that it is of an acceptable scientific standard.

# Reviewer report: Reviewer response for version 2

**DOI:** 10.21956/openreseurope.21853.r54001

**Published:** 2025-05-28

**Authors:** Pedro Chamusca

**Affiliations:** 1 University of Minho, University of Minho, Portugal

The authors have carefully addressed all the suggestions and recommendations provided in the first review round. The revised version reflects significant improvements in both structure and content, resulting in a more robust and engaging article. In light of these enhancements, the manuscript is now suitable for indexing.

If applicable, is the statistical analysis and its interpretation appropriate?

Not applicable

Is the study design appropriate and is the work technically sound?

Partly

Is the work clearly and accurately presented and does it engage with the current literature?

Partly

Are the conclusions drawn adequately supported by the results?

Partly

Are sufficient details of methods and analysis provided to allow replication by others?

Partly

Are all the source data and materials underlying the results available?

Partly

Reviewer Expertise:

Territorial cohesion; Regional and Local Development; Participation; Governance

I confirm that I have read this submission and believe that I have an appropriate level of expertise to confirm that it is of an acceptable scientific standard.

# Reviewer report: Reviewer response for version 1

**DOI:** 10.21956/openreseurope.20804.r52088

**Published:** 2025-04-08

**Authors:** María Mercedes Di Virgilio

**Affiliations:** 1 Instituto de Investigaciones Gino Germani, University of Buenos Aires | CONICET, Ciudad Autonoma de Buenos Aires, Ciudad Autónoma de Buenos Aires, Argentina

The article addresses territorial inequalities in Europe, intensified due to the lack of social protection and unequal access to essential services. In this frame, based on a qualitative study, the paper explores stakeholders' perceptions of the underlying drivers of territorial inequalities, the governance mechanisms, and the policy tools to mitigate them. The research was conducted in seven European countries: Austria, Belgium, Denmark, Greece, Italy, Serbia, and Spain. The article is based on 20 focus groups with 98 participants, including representatives from different levels of government and civil society organizations. This approach allows authors to capture local experiences and perceptions of territorial inequalities.

 Research findings indicate a disconnection between national policies and the priorities of local actors. Governance challenges, such as fragmentation and lack of cooperation, hinder the reduction of territorial inequalities. The article highlights the need for specific, context-sensitive approaches to address territorial disparities. It concludes that policies that consider local realities and promote cooperation between different levels of government are essential. Interventions must be aligned with the specific needs of the areas “left behind” to mitigate territorial disparities effectively.

 Using focus groups provides an in-depth understanding of local experiences and stakeholder perceptions. Likewise, including seven European countries allows for a rich and varied comparison of perceptions of territorial inequalities. Finally, the study addresses a critical issue in European policy, which can inform future decisions and improve the effectiveness of cohesion policies.

 Although the article extensively reviews the literature discussing place-sensitive policy approaches, it could benefit from a more in-depth review of the existing literature on territorial inequalities, especially on the production of inequalities in contexts where regions and local governments have high levels of autonomy. Especially in the literature concerned with producing inequalities in contexts where regions and local governments have high levels of territorial autonomy. How do disparities in local economies play a role in the production of territorial inequalities? How do characteristics of the population living in cities influence the mechanisms of resource sharing and redistribution that mediate relations between central and subnational governments?

 The selection criteria for the countries participating in the study, the justification of the participants who integrated the groups, and the number of focus groups are not well specified. It would be important to have a profile of the group participants: position, age, sex, education, etc.

 More information on the political organization of the countries under analysis, the competencies of local governments, and their levels of autonomy would strengthen the research findings. This information would allow a better contextualization and understanding of the study findings.

 The article offers valuable insights into territorial inequalities in Europe, but it could be improved regarding the clarity of its theoretical framework and the depth of its interpretations. The research highlights the need for more integrated and context-sensitive approaches to address territorial disparities effectively.

If applicable, is the statistical analysis and its interpretation appropriate?

Not applicable

Is the study design appropriate and is the work technically sound?

Partly

Is the work clearly and accurately presented and does it engage with the current literature?

Partly

Are the conclusions drawn adequately supported by the results?

Yes

Are sufficient details of methods and analysis provided to allow replication by others?

No

Are all the source data and materials underlying the results available?

Yes

Reviewer Expertise:

Public policies, urban inequalities.

I confirm that I have read this submission and believe that I have an appropriate level of expertise to confirm that it is of an acceptable scientific standard, however I have significant reservations, as outlined above.

# Response

**Published:** 2025-04-22

**Authors:** Malin Roiha

**Affiliations:** Departament d'Antropologia Social, Universitat de Barcelona Departament de d'Antropologia Social, Barcelona, Catalonia, Spain

Thank you for your detailed and thoughtful feedback on our article. We greatly appreciate the time and care you dedicated to engaging with our work. Your insights have been invaluable in helping us enhance the clarity and focus of the paper. In response to your comments, as well as those from other reviewers, we have revised several key aspects, which are now reflected in Version 2. The main changes in relation to your specific feedback are as follows: We have provided a clearer rationale for the selection of countries and research areas within those countries. Additionally, we have included more detailed information on the composition of the focus group sample, including demographics and representation across governance levels and sectors, to the extent that this data was collected during the research. Furthermore, we have strengthened our engagement with the literature, particularly in the conclusion section

# Reviewer report: Reviewer response for version 1

**DOI:** 10.21956/openreseurope.20804.r51802

**Published:** 2025-03-18

**Authors:** Pedro Chamusca

**Affiliations:** 1 University of Minho, University of Minho, Portugal

The article "Between Policy and Perception: Stakeholder Views on Addressing Territorial Inequality in Europe" provides a compelling examination of territorial disparities through a governance lens, highlighting the divergence between national policies and local stakeholder perspectives. By drawing on focus group discussions across seven European countries, the study offers valuable qualitative insights into policy implementation challenges and the factors that contribute to regional inequalities. The work has strong potential to inform policy debates, particularly regarding the effectiveness of EU cohesion policies and place-sensitive governance approaches. Its emphasis on qualitative narratives adds depth to discussions on territorial inequality, moving beyond macroeconomic indicators to explore lived experiences and governance constraints.

 Despite its strengths, the article would benefit from a clearer articulation of its theoretical contributions, enhanced methodological transparency, and more concrete policy recommendations. Addressing these areas will significantly improve its impact and applicability for policymakers, researchers, and regional development practitioners. Strengthening the integration of literature, refining the methodological framework, and ensuring accessibility of data will help bridge the gap between empirical findings and actionable policy insights. This evaluation provides detailed suggestions to enhance the clarity, coherence, and rigor of the article, ensuring its contributions to the field of territorial inequality research are maximized.

**SPECIFIC CRITERIA**

**1. Is the work clearly and accurately presented and does it engage with the current literature? (Partly)**

 The article presents a well-structured and comprehensive discussion on territorial inequalities in Europe, integrating qualitative insights from stakeholders. The theoretical framework is grounded in recent literature on territorial disparities and governance challenges, referencing key scholars such as Rodríguez-Pose, MacKinnon, and Crescenzi. However, the engagement with literature could be expanded in two ways: A clearer synthesis of how existing theories on territorial inequality and governance relate to the findings; and a deeper discussion on the nuances of "left-behind places" in different socioeconomic and political contexts, considering the specificities of various EU cohesion policies.

**2. Is the study design appropriate and is the work technically sound? (Partly)**

 The study employs qualitative methods through focus groups, a suitable approach for capturing subjective experiences of governance mechanisms and policy implementation. The cross-country comparison provides valuable insights into variations in territorial inequality governance. However, certain limitations should be addressed:

- The selection criteria for focus group participants could be more explicitly justified to ensure representation across different governance levels and sectors.
- The rationale behind the number of focus groups (20) and sample size (98 participants) requires further elaboration to clarify how it ensures data saturation and validity.
- Some descriptions of qualitative coding and analysis are vague. More details on the coding framework, thematic analysis, and reliability measures would enhance methodological robustness.

**3. Are sufficient details of methods and analysis provided to allow replication by others? (Partly)**

 While the methodological section provides a general overview of data collection and analysis, it lacks specific details necessary for replication, such as: the full list of guiding questions used in focus groups; more precise information on data processing, including inter-coder reliability assessments and validation steps; and the rationale for selecting the seven European countries and how their inclusion ensures a balanced perspective on territorial disparities.

**4. Are all the source data and materials underlying the results available? (Partly)**

 The study states that focus group data is stored in a restricted-access Zenodo repository, requiring approval for access. While this ensures data confidentiality, a more transparent approach—such as anonymized excerpts or coded summary tables—could strengthen the credibility of findings. Additionally, a more detailed description of the dataset (e.g., demographics of participants, institutional affiliations) would improve reproducibility.

**5. If applicable, is the statistical analysis and its interpretation appropriate? (Not applicable)**

**6. Are the conclusions drawn adequately supported by the results? (Partly)**

 The conclusions align with the study's findings, emphasizing governance fragmentation, policy centralization vs. decentralization, and funding disparities. However, it would be strengthened with a more explicit discussion on policy recommendations, with concrete proposals for overcoming governance barriers, as well as a critical reflection on the limitations of the study and potential biases in stakeholder perspectives.

 The article provides valuable insights into the governance challenges of territorial inequality in Europe, with a well-organized qualitative analysis. However, to enhance its scientific rigor and impact, the authors should strengthen engagement with existing literature, improve methodological transparency, provide clearer policy recommendations based on empirical findings and address limitations and future research directions. If these improvements are made, the article will be significantly strengthened in its contribution to territorial governance research.

 Thus, the authors should revise and resubmit with major revisions, considering:

1. Enhancing theoretical engagement
2. Strengthening methodological transparency
3. Improving data accessibility and presentation
4. Clarifying policy implications and recommendations

If applicable, is the statistical analysis and its interpretation appropriate?

Not applicable

Is the study design appropriate and is the work technically sound?

Partly

Is the work clearly and accurately presented and does it engage with the current literature?

Partly

Are the conclusions drawn adequately supported by the results?

Partly

Are sufficient details of methods and analysis provided to allow replication by others?

Partly

Are all the source data and materials underlying the results available?

Partly

Reviewer Expertise:

Territorial cohesion; Regional and Local Development; Participation; Governance

I confirm that I have read this submission and believe that I have an appropriate level of expertise to confirm that it is of an acceptable scientific standard, however I have significant reservations, as outlined above.

# Response

**Published:** 2025-04-22

**Authors:** Malin Roiha

**Affiliations:** Departament d'Antropologia Social, Universitat de Barcelona Departament de d'Antropologia Social, Barcelona, Catalonia, Spain

Thank you for your detailed and thoughtful feedback on our article. We sincerely appreciate the time and care you took in engaging with our work. Your insights have been invaluable in helping us refine the clarity and focus of the paper. Based on your comments, we have revised several key aspects, which are now reflected in Version 2. The main changes in relation to your specific comments are as follows: We have strengthened the engagement with relevant literature, particularly in the conclusions section. Further, we have rewritten the methods section to reflect the demographics and representation of focus groups across governance levels and sectors; the rationale behind the number of focus groups and sample size; and more details on the coding framework. Additionally, more information on the sample has been included in the description of the Zenodo dataset. The full list of the guiding questions used in the focus group discussions is also available as extended data on Zenodo. Finally, the conclusions now include a more explicit discussion of policy implications, offering concrete proposals to address governance barriers. We have also added a critical reflection on the study’s limitations.

# Reviewer report: Reviewer response for version 1

**DOI:** 10.21956/openreseurope.20804.r52091

**Published:** 2025-03-17

**Authors:** Ray Hudson

**Affiliations:** 1 University of Durham, England, UK

This paper addresses important issues in relation to uneven development and inequality in the EU and the diverse processes of governance and regulation through which the EU, national states and various sub-national organisations seek to address and contain the resultant tensions and pressures. The authors locate this in relation to recent literature on ‘left behind places’ by way of providing a context for their approach. Information as to the criteria for the choice of national and places within them, alongside some statistical data that would have helped provide a context for their approach to the choice of places, since places get ‘left behind’ for diverse reasons, with implications as to what might b appropriate policies to enable them to ‘catch up’. However, to talk about the interests of ‘places’ without unpacking *who* in a place will benefit from particular policy approaches, *who* will not, and why, can be problematic. The benefits of any given policy approaches will be unevenly distributed and it is important to clarify who ‘wins’ and who ‘loses out’ as a result of pursuing a particular policy approach. Their approach is essentially descriptive and this is appropriate in terms of scoping the variety of perceptions of the problem and policy responses to it. In principle, a methodological approach that incorporates focus groups as a source of evidence appropriate but I have a number of reservations and questions about their methodological approach. For example, there is little information as to why the groups had so few members, as to who the members of the groups were and as to their position in relevant places and organisations. Some clarification on these issues would strengthen the paper and allow more considered conclusions as to appropriate policies for these diverse places.

If applicable, is the statistical analysis and its interpretation appropriate?

Not applicable

Is the study design appropriate and is the work technically sound?

Partly

Is the work clearly and accurately presented and does it engage with the current literature?

Partly

Are the conclusions drawn adequately supported by the results?

Partly

Are sufficient details of methods and analysis provided to allow replication by others?

Partly

Are all the source data and materials underlying the results available?

Partly

Reviewer Expertise:

Political economy and political and economic geogr

I confirm that I have read this submission and believe that I have an appropriate level of expertise to confirm that it is of an acceptable scientific standard, however I have significant reservations, as outlined above.

# Response

**Published:** 2025-04-22

**Authors:** Malin Roiha

**Affiliations:** Departament d'Antropologia Social, Universitat de Barcelona Departament de d'Antropologia Social, Barcelona, Catalonia, Spain

Thank you for your detailed and thoughtful feedback on our article. We genuinely appreciate the time and care you invested in engaging with our work. Your insights have been invaluable in helping us refine both the clarity and focus of the paper. In response to your comments, we have made several revisions, which are now reflected in Version 2. The main changes related to your specific feedback are as follows: We have provided a clearer rationale for the selection of countries and research areas. Additionally, we have added more detail on the composition of the focus group sample, including reflections on the group sizes. Regarding your comment on identifying who in a place will benefit from particular policy approaches, based on the data analysed in this paper, we are not able to include a discussion on this specific point. However, this issue has been explored in greater depth, particularly from an intersectional perspective, in other parts of the research, mainly through ethnographic fieldwork.  The consortium is currently developing several papers in relation to this data.
